# Influence of Phytase Transgenic Corn on the Intestinal Microflora and the Fate of Transgenic DNA and Protein in Digesta and Tissues of Broilers

**DOI:** 10.1371/journal.pone.0143408

**Published:** 2015-11-23

**Authors:** Lin Lu, Jiang Guo, Sufen Li, Ang Li, Liyang Zhang, Zhenhua Liu, Xugang Luo

**Affiliations:** 1 Mineral Nutrition Research Division, Institute of Animal Sciences, Chinese Academy of Agricultural Sciences, Beijing, China; 2 College of Animal Science, Fujian Agriculture and Forestry University, Fuzhou, China; 3 Department of Animal Science, Hebei Normal University of Science and Technology, Qinhuangdao, China; 4 Department of Nutrition, School of Public Health and Health Sciences, University of Massachusetts, Amherst, Massachusetts, United States of America; Huazhong University of Science and Technology, CHINA

## Abstract

An experiment was conducted to investigate the effect of phytase transgenic corn (PTC) on intestinal microflora, and the fate of transgenic DNA and protein in the digesta and tissues of broilers. A total of 160 1-day-old Arbor Acres commercial male broilers were randomly assigned to 20 cages (8 chicks per cage) with 10 cages (replicates) for each treatment. Birds were fed with a diet containing either PTC (54.0% during 1–21 days and 61.0% during 22–42 days) or non-transgenic isogenic control corn (CC) for a duration of 42 days. There were no significant differences (P>0.05) between birds fed with the PTC diets and those fed with the CC diets in the quantities of aerobic bacteria, anaerobic bacteria, colibacillus and lactobacilli, or microbial diversities in the contents of ileum and cecum. Transgenic *phyA2* DNA was not detected, but *phyA2* protein was detected in the digesta of duodenum and jejunum of broilers fed with the PTC diets. Both transgenic *phyA2* DNA and protein fragments were not found in the digesta of the ileum and rectum, heart, liver, kidney, and breast or thigh muscles of broilers fed with the PTC diets. It was concluded that PTC had no adverse effect on the quantity and diversity of gut microorganisms; Transgenic *phyA2* DNA or protein was rapidly degraded in the intestinal tract and was not transferred to the tissues of broilers.

## Introduction

Phosphorus (P) sources in the world are limited, and overconsumption of P could result in a crisis in P supply [1]. However, phytate-P is poorly available to poultry from their diet [2], due to the low activity of phytase in their digestive tracts [3], and thereby inorganic P must be supplemented in poultry diets to meet their P requirements. Consequently, the unabsorbed phytate-P is leading to a serious environmental problem. To manage this problem, the microbial phytase is added to diets routinely to improve P utilization [4, 5]. However, this approach has the following disadvantages: 1) it requires a high productive cost of the enzyme and a special care in feed formulation and processing [6, 7]; 2) the phytase needs to be added to the premixes containing some other components (such as copper sulfate, etc) that may affect its stability [8, 9]; and 3) the phytase and the corn need to be purchased separately, and then mixed that will cost time and labor [10]. Therefore, the production and application of plant seeds containing high phytase activities could be selected as an alternative approach.

It has been shown that the phytases produced in some plants could substitute for the phytases produced from microbial fermentation [11, 12]. Phytase transgenic corn (PTC), which expresses a phytase gene (*phyA2*) from Aspergillus niger [6], can increase the bioavailability of P, and thereby the need of inorganic P would be reduced in the diet, resulting a reduction of P excretion [7, 10]. In addition, the residual activity of the transgenic corn-derived phytase was higher than those of commercial microbial phytases in the crop and gastrointestinal tract (GIT) of poultry [13].

The PTC, in which a microbial-origin gene is inserted, is extremely promising in terms of environmental health [6]. However, public concerns have been raised in regard to the usage of genetically modified (GM) crops and their potential effects on animal and human health [14]. Therefore, it is essential to evaluate the fates of transgenic DNA and protein from the GM crop in animals before the crop can be introduced to the feed market. Some studies have been conducted to examine the residuals of genetically engineered components in the organs/tissues of animals fed with GM crops. Ma *et al*. [15] reported that the transgenic *phyA2* gene and protein were detected in the digesta of upper GIT, but not detected in the blood, tissues, and eggs of laying hens fed with PTC. Chowdhury *et al*. also demonstrated that Cry1Ab protein was mostly degraded in the GIT, and not retained in the liver, spleen, kidney, lymph nodes or muscles of pigs [16] Similar findings with Bt 176 maize and Roundup Ready Soybean meal were reported by Tony *et al*. [17] and Jennings *et al*. [18], respectively. Up to now, no data are available on the fates of phytase transgenic DNA and protein in the digesta and tissues of broilers. Moreover, animal diets have critical impacts on both the quantity and composition of microbes in intestinal microflora [19, 20]**,** but the effect of PTC on intestinal microflora of broilers has not been investigated.

The objective of this study was to address the effect of PTC on intestinal microbial microflora, and to determine if the *phyA2* gene or protein is retained in the digesta and tissues of broilers after consumption.

## Materials and Methods

This study was reviewed and approved by the Animal Welfare Committee of the Institute of Animal Sciences, Chinese Academy of Agricultural Sciences. All experimental procedures were performed according to the principles and guidelines for the Care and Use of Animals of the Chinese Academy of Agricultural Sciences.

### Corns and diets

The PTC and non-transgenic isogenic control corn (CC) were the same as those used in the study of Ma *et al*. [15] or Li *et al*. [7]. To avoid potential contamination, the control diet was mixed first, and then followed by the PTC diet. Nutrient levels of both diets were analyzed using the procedure of Gao *et al*. [10]. Phytase activity in the diet was measured as described by Engelen *et al*. [21]. One phytase activity unit (FTU) was defined as the amount of activity that liberates 1μM of inorganic P per min from 1.5 mM sodium phytate at pH 5.5 at 37°C. The two treatment diets were formulated to meet the requirements of broilers recommended by the National Research Council (Table 1) [22].

**Table 1:** Composition of the diets (as-fed basis).

Item (% unless note)	Days 1–21		Days 22–42	
	CC^1^	PTC^1^	CC^1^	PTC^1^
**Ingredient**				
**Corn (non-GM)**	54.00		61.00	
**Corn (GM)**		54.00		61.00
**Soybean meal (non-GM)**	37.50	37.50	30.60	30.60
**Corn oil (non-GM)**	3.80	3.35	4.00	3.10
**Limestone**	1.40	1.50	1.50	1.72
**Dicalcium phosphate**	1.98	1.81	1.35	0.95
**Salt**	0.30	0.30	0.30	0.30
**DL-Methionine**	0.22	0.22	0.10	0.10
**Micronutrients** ^2^	0.33	0.33	0.23	0.23
**Corn starch**	0.47	0.99	0.92	2.00
**Nutrient composition**				
**ME(MJ/kg)**	12.39	12.44	12.80	12.77
**CP** ^3^	21.94	21.97	19.17	19.23
**Lysine**	1.15	1.13	1.00	1.00
**Methionine**	0.56	0.56	0.41	0.41
**Methionine +cystine**	0.91	0.89	0.74	0.72
**Ca** ^3^	1.03	1.03	0.88	0.92
**Total P** ^3^	0.78	0.73	0.58	0.53
**Nonphytate P** ^3^	0.46	0.44	0.40	0.40
**Phytase activity** ^3^ **(FTU/Kg)**	68.04	4314.99	50.08	5113.16

^1^CC = control corn; And PTC = phytase transgenic corn.

^2^For starter diets, provided per kilogram of diet: vitamin A 12500 IU, vitamin D_3_ 3750 IU, vitamin E 20 IU, vitamin K 2.5 mg, thiamine 2.5 mg, riboflavin 8 mg, pyridoxine 2.5 mg, vitamin B_12_ 0.015 mg, cacium pantothenate 12.5 mg, niacin 32.5 mg, folic acid 1.25 mg, biotin 0.125mg, choline 700 mg, zinc 60 mg, copper8 mg, iron 80 mg, manganese 100 mg, iodine 0.35 mg, and selenium 0.15 mg. For grower diets, provided per kilogram of diet: vitamin A 10000IU, vitamin D_3_ 3000IU, vitamin E 16IU, vitamin K 2mg, thiamine 1 mg, riboflavin 6.4mg, pyridoxine 2mg, vitamin B12 0.012mg, cacium pantothenate 10mg, Niacin 26mg, folic acid 1mg, biotin 0.1mg, choline 500mg, zinc 40 mg, copper 8mg, iron 80 mg, manganese 80 mg, iodine 0.35 mg, and selenium 0.15 mg.

^3^Determined by analysis.

### Animal housing and management

One hundred and sixty 1-day-old Arbor Acres commercial male broilers were assigned to 1 of 10 replicate cages (8 chicks per cage) for each of 2 treatments in a completely randomized design. Experimental diets contained either CC or PTC (54.0% during 1 to 21 days and 61.0% during 22 to 42 days). Broilers were raised in stainless cages coated with plastic (100×50×45 cm) and equipped with fiberglass feeders and waterers. During 42 days, birds were allowed *ad libitum* access to experimental diets and tap water, and maintained on a 24-h light schedule. The management of the birds was in accordance with guidelines approved by Arbor Acres Breeding Company in Beijing.

### Sample collections

At 42 days of age, 2 birds from each cage (20 birds/group) were sacrificed by cutting the carotid arteries and bled after a 12-h fast. The heart, liver, kidney, breast and leg muscles were removed, and the contents of rectum, ileum, jejunum, and duodenum were also collected for DNA and protein analysis. At the same time, 2 birds from each cage (20 birds/group) were anesthetized by intravenous injection of Sumianxin II, and then killed by cutting the carotid arteries and bled. Ileal and cecal contents of the birds were collected under sterile conditions for the measurement of microbial population and diversity. All the above samples were snap-frozen in liquid nitrogen and then frozen at –80°C until analysis.

### Incubation and enumeration of intestinal microorganisms

After the intestinal samples were thawed at 4°C, one gram of the ileal or cecal contents was diluted with 9 mL of sterile water and mixed on a vortex. The suspensions were diluted by 10-7-times, and then the dilutions were plated on nutrient agar medium, eosin methylene blue medium, GMA broth medium and LBS medium for the culture of aerobic bacteria, colibacillus, anaerobic bacteria and lactobacilli, respectively. All the media were obtained from Beijing Land Bridge Technology Co., Ltd. Aerobic bacteria and colibacillus were cultured in an incubator (HPS-250, Harbin, P. R. China) at 37°C for about 30 h. Anaerobic bacteria and lactobacilli were cultured in an anaerobic (28% CO2, 15% N2, and 25%H_2_) incubator (YQX-2, Shanghai, P. R. China) at 37°C for about 48 h. Viable counts of microbials in the ileal and cecal samples were then conducted immediately after removal from the incubator.

### 16S rRNA PCR-denaturing gradient gel electrophoresis (DGGE) analysis

#### Genomic DNA extraction

Genomic DNA was extracted from ileal and cecal samples using QIAamp DNA Stool Mini Kit (Qiagen GmbH, Hilden, Germany) following the manufacturer’s protocol, and then stored at –20°C.

#### PCR

The V3 region of 16S rDNA was amplified by PCR using a pair of universal primers designed by Aoke Dingsheng Bio-tech (Beijing) Company, Limited. The oligonucleotides used were as follows: the upstream primer was 339F (5’-CGCCCGGGGCGCGCCCCGGGCGGGGCGGGGGCACGGGGGGACTCCTACGGGAGGCAGC-3’); and the downstream primer was 539R (5’-GTATTACCGCGGCTGCTGGCA-3’) [23].

PCR amplification was performed in a 50-μL reaction volume as follows: 1μL of the template DNA (20–100 ng/μL), 1 μL of each primer (10 μM), 25 μL of 2×Reaction Mix (2.5 mM; Tiangen Biotech. Co. LTD., Beijing, China), 0.5 μL of Tag polymerase (4 U/μL), and 21.5 μL of ddH_2_O.

The reaction was denatured at 94°C (4 min), followed by 35 cycles of 30s at 94°C, 30s at 56°C, 1 min at 72°C, and an extension at 72°C (10 min).

#### DGGE

DGGE was performed using the method as previously described [23]. Up to 35% to 65% linear DNA-denaturing gradients were formed in [15]10% polyacrylamide gels. Low and high concentrations of the denaturing gradient gel solutions were then prepared (Table 2). Bacterial V_3_ 16S PCR products were loaded in each lane, and electrophoresis was conducted in 1× TAE Buffer at a voltage of 200 V at 60°C for 10 min, and then at 80 V for 16 h. After electrophoresis, the gels were silver-stained and scanned using a GS-800 Calibrateda Densitometer system [24, 25].

**Table 2:** Composition and content of the denaturing gradient gel solution^1^.

Composition	35% Denaturant (LOW)	65% Denaturant (HIGH)
**40% (W/V)Acrylamide/Bisacrylamide**	25 mL	25 mL
**50xTAE Buffer**	2 mL	2 mL
**Deionized Formamide**	14 mL	26 mL
**Urea**	14.7 g	27.3 g
**ddH**_**2**_ **0**	To100 mL	To100 mL

^1^The 100% denaturant is equivalent to 7 mol/L of urea and 40% (v/v) deionized formamide.

### DNA analysis

#### DNA extraction

DNA from the CC, PTC, diets, intestinal contents, or tissues was extracted using the method reported by Ma et al. [15], and then stored at -80° C.

#### PCR analysis

The *ivr* (a specific invertase gene of corn, 226 bp) and *phyA2* (a foreign gene in the PTC, 678 bp) were used to monitor corn DNA and transgenic DNA, respectively. Furthermore, the chicken ovalbumin gene (*ov*, 396 bp) in tissues was determined to validate the DNA extraction for PCR. The *ivr*, *phyA2* and *ov* genes were acquired from AuGCT, Beijing, China. The primers and PCR conditions were showed in a previous study [15]. Separation of products of the PCR and analysis of the gel images were performed according to the method as previously described [15].

### Western blot analysis

Samples from the corn, diets, digesta and tissues were homogenated and cleaved using RIPA lysis buffer (Beyotime Institute of Biotechnology, Beijing, P. R. China). After centrifuged at 12,000×g for 5 min at 4°C, the supernatants were collected for SDS-PAGE and western blotting analysis. Protein concentrations were determined by Bradford Protein Assay Kit (Beyotime Institute of Biotechnology, Beijing, P. R. China). Other procedures for western blot analysis were carried out as described by Ma *et al*. [15].

### Statistical analyses

The data were analyzed by one-way ANOVA using the GLM procedure of SAS (SAS Institute, Inc., 2001). Differences between the two treatments were analyzed using t-tests. Differences were considered significant if *P* < 0.05. The data were shown as mean ± SD.

## Results

### Influence of PTC on microbial population in the intestine of broilers

There were no significant differences (P>0.05) between the birds fed with the PTC and CC diets in the populations of aerobic bacteria, anaerobic bacteria, colibacillus, or actobacilli in ileal or cecal contents (Table 3).

**Table 3:** Effect of phytase transgenic corn on microbial counts in the ileal and cecal contents of broilers (lg cfu·g-1)^1^.

Intestinal tract	Item	N	CC	PTC	P-value
**Ileum**	Aerobic bacteria	20	6.61±0.20	6.65±0.17	0.90
	Anaerobic bacteria	20	6.49±0.15	6.64±0.14	0.50
	Colibacillus	20	5.70±0.17	5.62±0.16	0.74
	Lactobacilli	20	6.18±0.12	6.43±0.18	0.25
**Cecum**	Aerobic bacteria	20	7.03±0.19	7.02±0.18	0.97
	Anaerobic bacteria	20	7.14±0.15	7.10±0.18	0.88
	Colibacillus	20	6.54±0.15	6.86±0.18	0.18
	Lactobacilli	20	6.61±0.18	6.84±0.19	0.40

^1^Data are expressed as mean ± SD (n = 20); And cfu means colony-forming unit.

### Microbial diversity in the intestine of broilers

**Fig 1:**
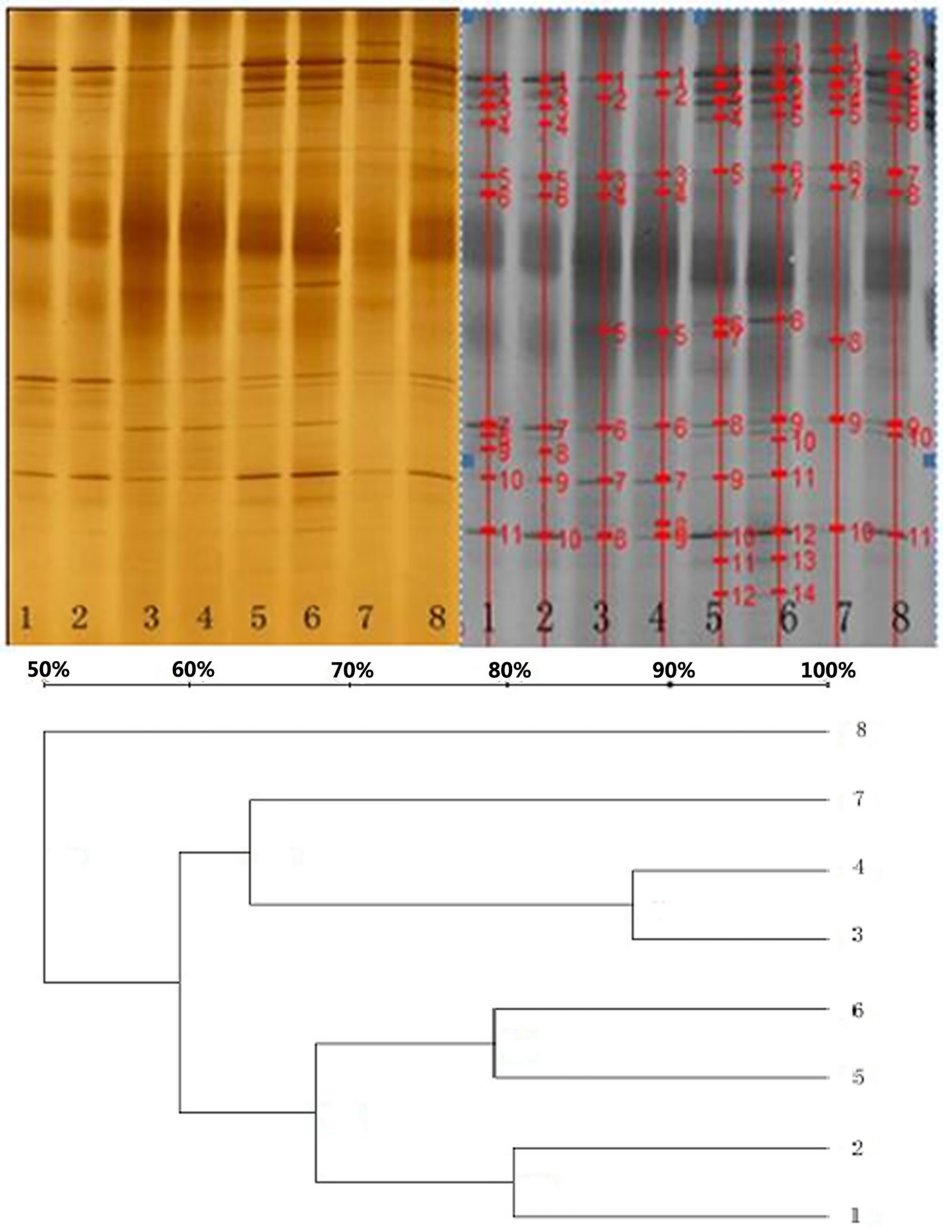
Denaturing gradient gel electrophoresis (DGGE) of ileal microbial 16S amplicon from broilers on phytase transgenic corn (PTC), and control corn (CC) diets. The left one was scanned by GS-800 Calibrated Densitometer system, and the right one was analyzed by Quantity One system. Lanes 1, 2, 5, and 6 were from ileum of PTC-fed birds, and lanes 3, 4, 7, and 8 were from ileum of CC-fed birds. Dendrogram illustrated the similarity of band patterns.

**Fig 2:**
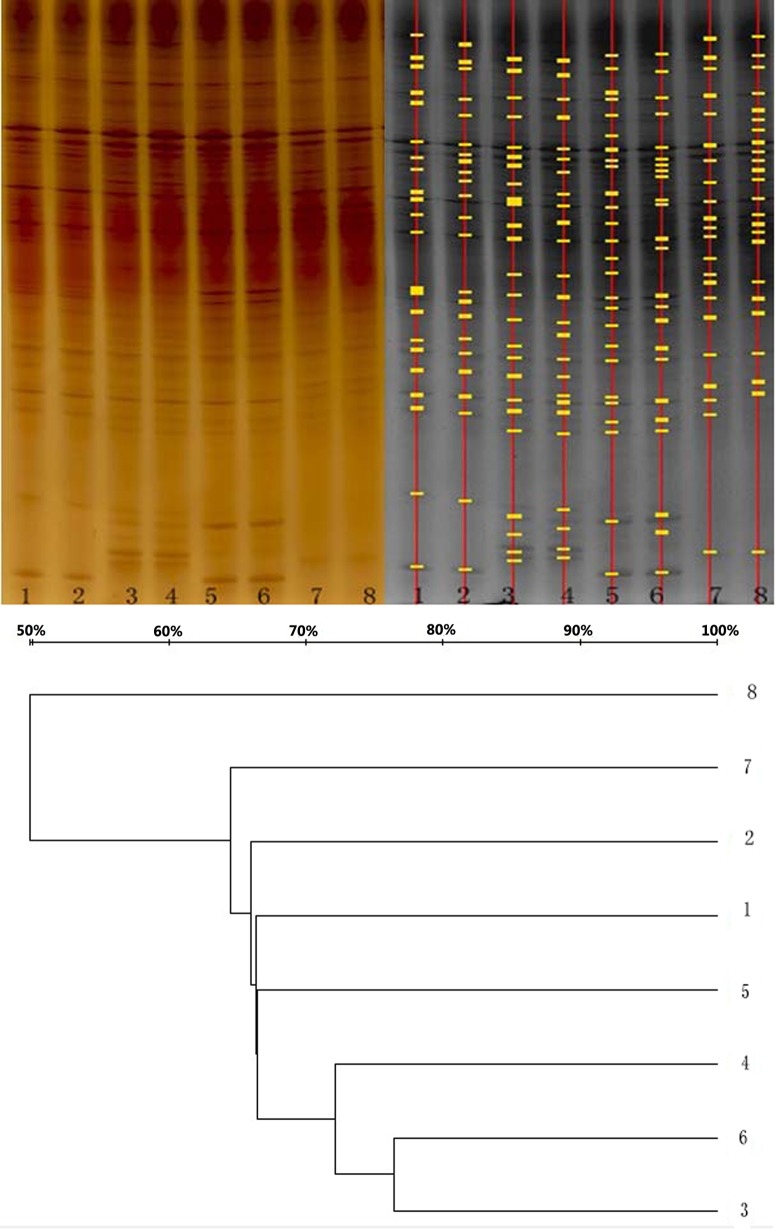
Denaturing gradient gel electrophoresis of cecal microbial 16S amplicon from broilers on control corn (CC), and phytase transgenic corn (PTC) diets. The left one was scanned by a GS-800 Calibrated Densitometer system, and the right one was analyzed by a Quantity One system. Lanes 1, 2, 5, and 6 were from the cecum of PTC-fed birds, and lanes 3, 4, 7, and 8 were from the cecum of CC-fed birds. Dendrogram illustrated the similarity of band patterns.

Birds fed with the PTC and CC diets had similar bands (Figs 1 and 2) in theirileum and cecum, and the band numbers in the ileum or cecum were also similar (P>0.05) (Table 4), indicating that dominant microfloras in the ileum or cecum were similar between the two treatments. In addition, the band numbers were higher (22.1 VS 10.4, P<0.05) in cecum than in ileum. This represents the diversity of microflora in different segments of the intestinal tracts of broilers.

**Table 4:** Effect of the phytase transgenic corn on the band numbers in ileum and cecum of broilers^1^.

Intestinal tract	N	CC	PTC	P-value
**Ileum**	20	10.05±0.35	10.18±0.47	0.84
**Cecum**	20	22.33±0.71	21.32±0.38	0.23

^1^Data are expressed as mean ± SD (n = 20).

The similarity coefficient (Cs) values of ileal microflora patterns for broilers fed with the same diets were numerically higher than those of cecal microflora patterns. Additionally, the Cs of ileal and cecal microflora patterns for broilers fed with different diets ranged from 59.4% to 34.6%, and 76.4% to 29.1%, respectively. However, different groups were not formed in the band patterns of ileum or cecum of birds fed with different diets. This suggested that PTC may not have an adverse effect on microflora in ileum and cecum.

### Detection of DNA fragment in corn, diets, digesta and tissues of broilers

**Fig 3:**
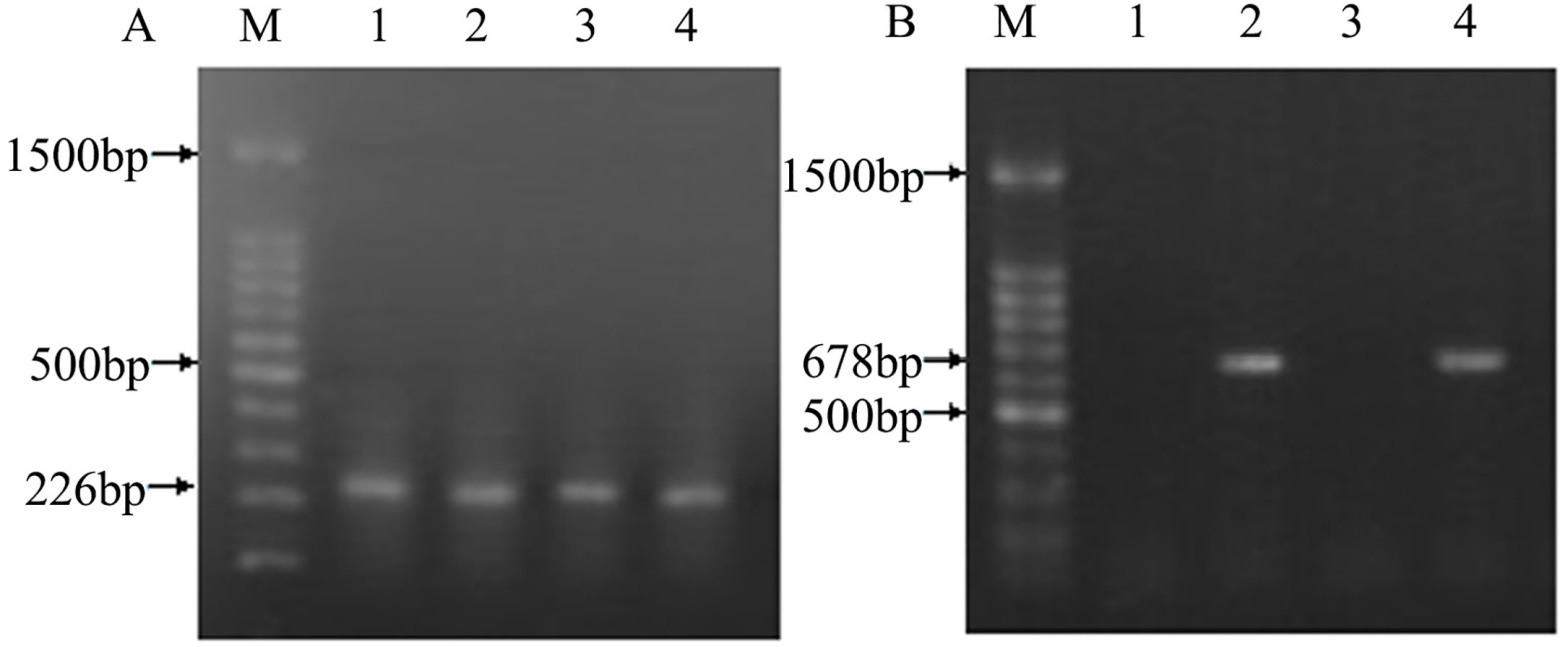
PCR amplification of genomic DNA from control corn (CC) and phytase transgenic corn (PTC). (A) PCR assay of the endogenous gene (*ivr*, 226 bp) of corn (left), and (B) PCR assay of the exogenous gene (*phyA2*, 678 bp) of PTC (right). M: marker;1: CC; 2: PTC;; 3: CC diet; And 4: PTC diet.

**Fig 4:**
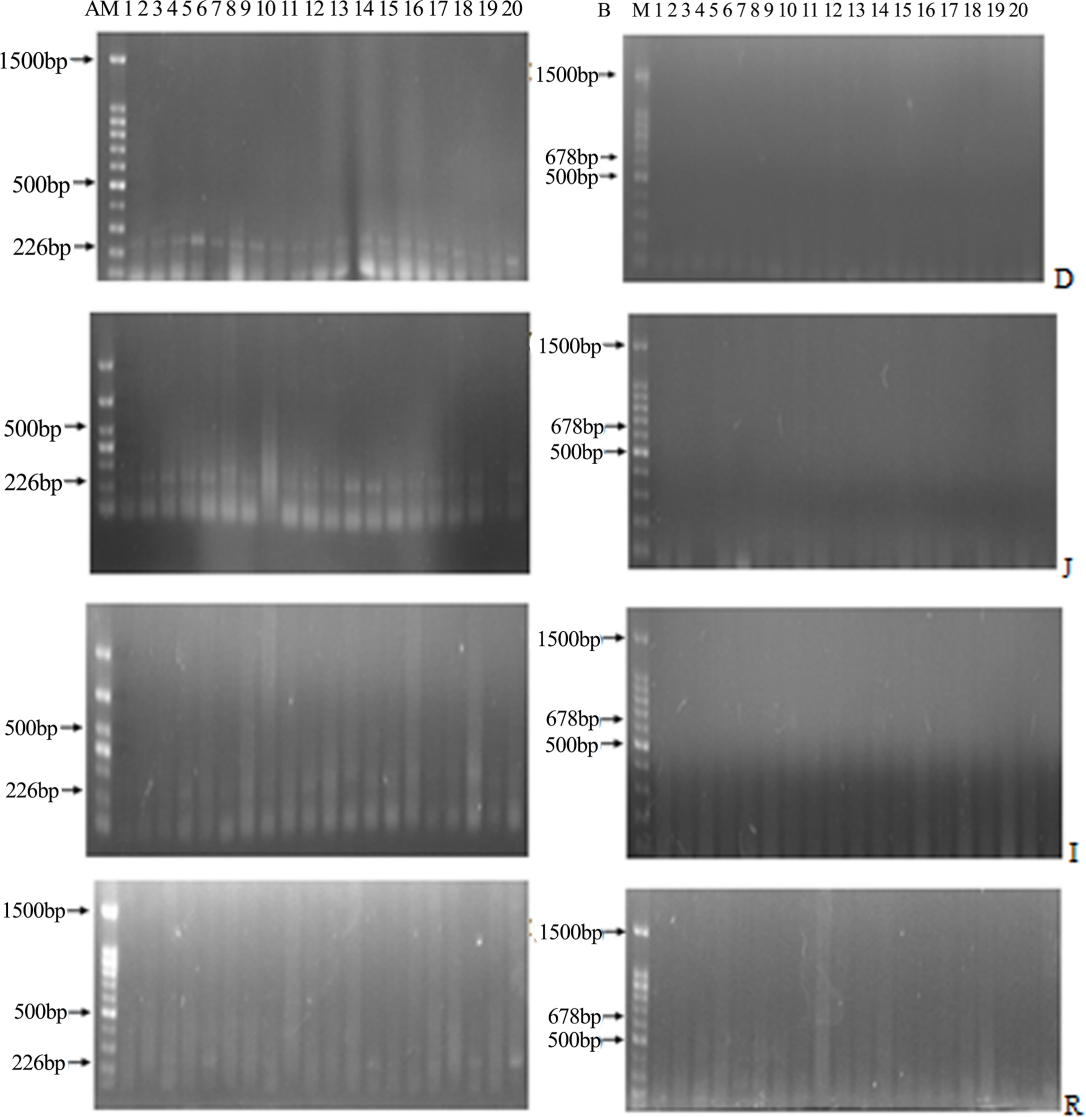
The PCR products from the digesta of broilers fed with either control corn (CC) or phytase transgenic corn (PTC). (A) PCR assay of the endogenous gene (*ivr*, 226 bp) of corn (left), and (B) PCR assay of the exogenous gene (*phyA2*, 678 bp) of PTC (right). M: marker; 1 to 10: CC-fed broilers; 11 to 20: PTC-fed broilers; D: duodenal contents; J: jejunum contents; I: ileum contents; and R: rectal contents.

**Fig 5:**
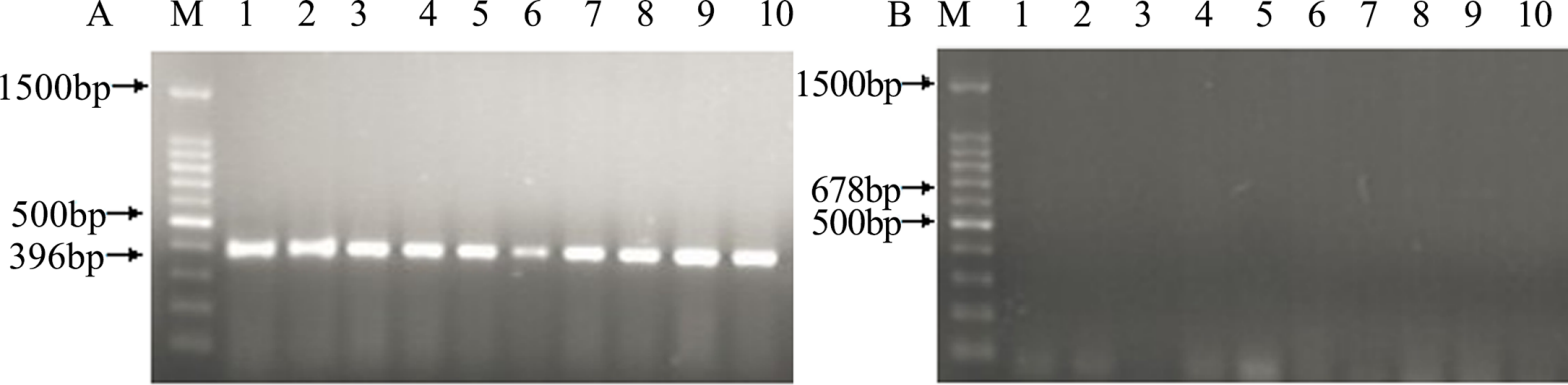
The PCR products from the tissues of broilers fed with either control corn (CC) or phytase transgenic corn (PTC). (A) PCR assay of the endogenous gene (*ov*, 396 bp) of chickens (left), and (B) PCR assay of the exogenous gene (*phyA2*, 678 bp) of PTC (right). Arrows represent the expected length of PCR products. M: marker; 2, 4, 6, and 8: heart, liver, kidney, breast muscle, and leg muscle of CC-fed broilers, respectively; And 3, 5, 7, 9, and 11: heart, liver, kidney, breast muscle, and leg muscle of PTC-fed broilers, respectively.

Fragments of the *ivr* gene were detected in all the corn and diet samples, whereas the transgenic *phyA2* gene fragments were only found in PTC and PTC diets (Fig 3). The *ivr* gene was also detected in all of the intestinal sectors from broilers fed with either a CC diet or a PTC diet. (Fig 4), whereas the *phyA2* gene was not detected in all of the intestinal sectors of broilers fed with the PTC diet (Table 5). The *ivr* gene was detected in a relatively high to low frequency as the extension of the intestine from broilers fed with both the CC and PTC diets (duodenum, 100% and 100%; jejunum, 80% and 90%; ileum, 50% and 60%; rectum, 10% and 20%, respectively). No transgenic *phyA2* gene fragments were detected in the heart, liver, kidney, breast muscle, and leg muscle (Fig 5, Table 6). Nevertheless, the endogenous chicken gene (*ov*) was detectable in all of the tissue of broilers fed with both the CC and PTC diets (Fig 5).

**Table 5:** Detections of endogenous and transgenic phyA2 gene in digesta of broilers fed either control corn (CC) or phytase transgenic corn (PTC)^1^.

Fragment amplified	Digesta^2^							
	duodenum		jejunum		ileum		rectum	
	-	+	-	+	-	+	-	+
***ivr***	10	10	8	9	5	6	1	2
***phy*A2**	0	0	0	0	0	0	0	0

^1^Number of samples that were positive for the gene of interest out of 10 samples analyzed. One sample was detemined per broiler (n = 10 broilers per treatment).

^2^‘‘-” represents CC-fed broilers, and ‘‘+” represents PTC-fed broilers.

**Table 6:** Detections of the transgenic *phyA2* gene in corns, diets, digesta, and tissues of broilers fed with either control corn (CC) or phytase transgenic corn (PTC) ^1^.

Item	Number of positive samples		Frequency of positive detection in CC- or PTC-fed broilers (%)^2^	
	CC	PTC	CC	PTC
**Corns**	0	10	0	100
**Diets**	0	10	0	100
**Duodenal contents**	0	0	0	0
**Jejunum contents**	0	0	0	0
**Ileum contents**	0	0	0	0
**Rectal contents**	0	0	0	0
**Heart**	0	0	0	0
**Liver**	0	0	0	0
**Kidney**	0	0	0	0
**Breast muscle**	0	0	0	0
**Leg muscle**	0	0	0	0

^1^Number of samples that were positive for the *phyA2* gene out of 10 samples analyzed. One sample was analyzed per broiler (n = 10 broilers per treatment).

^2^Percentage of samples containing the fragments of *phyA2* gene taken from PTC-fed broilers, i.e. (number of positive samples/number of samples analyzed) ×100.

### The phyA2 protein detection in corns, diets, digesta and tissues of broilers

**Fig 6:**
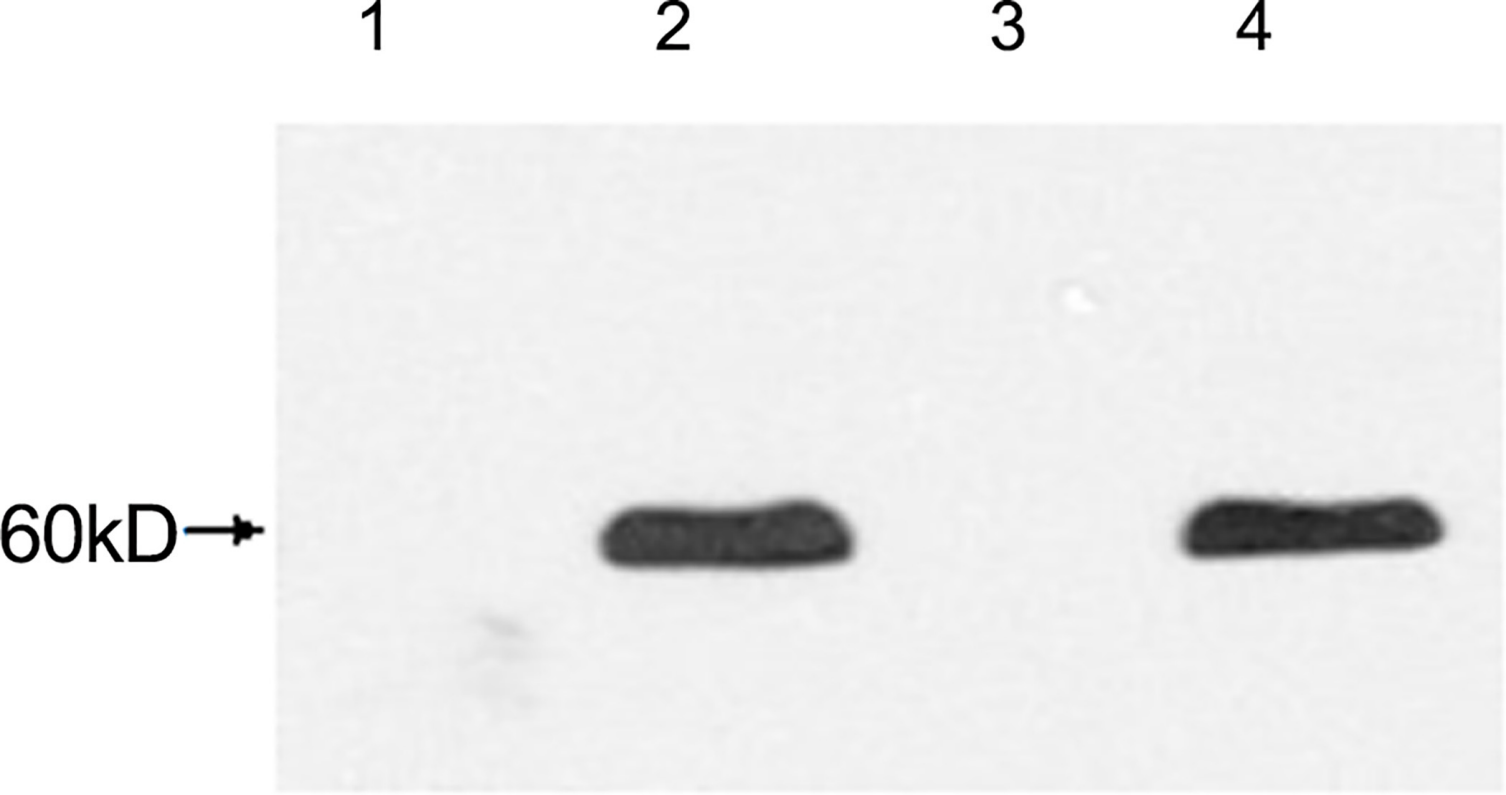
Western blot analysis of *phyA2* protein in corns and diets. 1: control corn (CC); 2: phytase transgenic corn (PTC); 3: CC diet; And 4: PTC diet.

**Fig 7:**
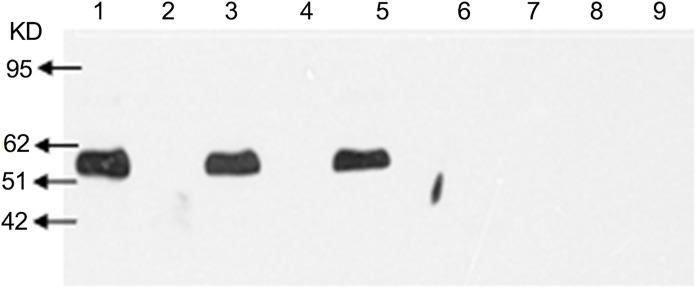
Western blot analysis of *phyA2* protein (60KD) in intestinal contents of broilers fed with either control corn (CC) or phytase transgenic corn (PTC). 1: phytase transgenic corn (PTC); 2, 4, 6, and 8: duodenal, jejunal, ileal, and rectal cotents of CC-fed broilers, respectively; And 3, 5, 7, and 9: duodenal, jejunal, ileal, and rectal contents of PTC-fed broilers, respectively.

**Fig 8:**
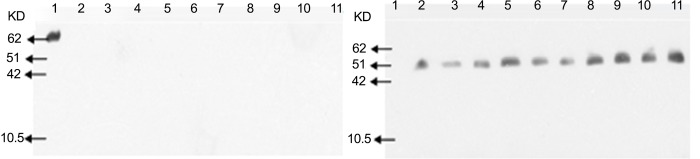
Western blot analysis of *phyA2* (left, 60KD) and β-actin (right, 43KD) protein in tissues of broilers fed with either control corn (CC) or phytase transgenic corn (PTC). 1: phytase transgenic corn (PTC); 2, 4, 6, 8, and 10: heart, liver, kidney, breast muscle, and leg muscle of CC-fed broilers, respectively; And 3, 5, 7, 9, and 11: heart, liver, kidney, breast muscle, and leg muscle of PTC-fed broilers, respectively.

*PhyA2* protein was not detected in all the CC and CC diet samples, but was shown in PTC and PTC diet samples (Fig 6). In addition, the *phyA2* protein was not observed in all the digesta samples from broilers fed with the CC diets (Fig 7). However, the *phyA2* protein was detected in the 70% of duodenum samples and 30% of jejunum samples taken from broilers fed with PTC (Table 7). The *PhyA2* protein was not detectable in the ileal and rectal contents from broilers fed with the PTC diets. The p*hyA2* protein was not detected in the heart, liver, kidney, breast and leg muscles of broilers fed with CC or PTC diets (Fig 8, Table 7), though the internal reference protein (β-actin) was detected in all the tissue samples of broilers fed with CC or PTC diets (Fig 8).

**Table 7:** Western blot results of the phyA2 protein in corns, diets, digesta, and tissues of broilers fed with either control corn (CC) or phytase transgenic corn (PTC) ^1^.

Item	Number of positive samples		frequency of positive detection in CC- or PTC-fed broilers (%)^2^	
	CC	PTC	CC	PTC
**Corns**	0	10	0	100
**Diets**	0	10	0	100
**Duodenal contents**	0	7	0	70.0
**Jejunum contents**	0	3	0	30.0
**Ileum contents**	0	0	0	0
**Rectal contents**	0	0	0	0
**Heart**	0	0	0	0
**Liver**	0	0	0	0
**Kidney**	0	0	0	0
**Breast muscle**	0	0	0	0
**Leg muscle**	0	0	0	0

^1^Number of samples that were positive for the phyA2 protein out of 10 samples analyzed. One sample was analyzed per broiler (n = 10 broilers per treatment).

^2^Percentage of samples containing the fragments of the phyA2 protein taken from broilers fed with PTC, i.e. (number of positive samples/number of samples analyzed) ×100.

## Discussion

### Influence of PTC on microbial microflora in the intestine of broilers

Intestinal microflora plays a critical role in the maintenance of health, the digestion, absorption, and metabolism of nutrients [26, 27]. Diet has significant impacts on the intestinal microbiota [28]. Plate culture is a traditional technique to detect intestinal microbial communities. The PCR-DGGE, a molecular biotechnology, provides a rapid, accurate and culture-independent survey of the microbial community, and has been widely used to study the gut microbiota of animals in the recent years. No significant differences in the number and diversity of intestinal microbes between broilers receiving non-GM diet and a GM rice-based diet have been reported [29]. Likewise, a previous report showed that there were no differences in the diversity of intestinal microbes between mice fed Bar-transgenic rice diet and conventional diet [30]. Similar findings in bees were also documented by some researchers [31, 32]. The results from the present study indicated that the number and diversity of intestinal microbes from broilers fed with PTC and CC diets were not different, indicating that PTC did not have an adverse effect on the intestinal microflora of broilers.

### Detection of phyA2 gene in digesta and tissues of broilers

Foreign DNA fragments are one concern in terms of safety associated with GM crops. Highly sensitive PCR can be used to detect transgenic DNA in animal tissues. The results from the current study showed that the *ivr* gene was detected with high efficiency in all the intestinal sectors of non-GM and GM treatments, and no significant differences on the *ivr* recovery in the gut were found between the two treatments. However, the *phyA2* D[20]NA was not detected in the different sections of intestine of the broilers fed with the PTC diets. Similar findings have been reported by Chambers *et al*. [33], who found that transgenic genes were not detectable in the distal GIT of chickens fed with GM corn. Ma *et al*. [15] also reported that fragments of the *phyA2* genes in the digesta of laying hens fed with a PTC diet reduced as the extension of the GIT (crop, 100%; gizzard, 87.5%; and small intestine or rectum, 0%). Thus, the transgenic phyA2 gene tends to be rapidly degraded as it moves through GIT of poultry. In addition, the *phyA2* gene was only detected in the PTC and PTC diets, and not in the CC and CC diets in this study, suggesting that cross-contamination between the two treatments was not found.

In general, some foreign DNA in feeds can be absorbed by animals. It is speculated that DNA of GM crops might transfer into tissues of domestic animals. It was reported that foreign DNA fragments could be inserted into immune cells of mice [34]. This makes it necessary to detect the fragments of foreign DNA in the gut and tissues of food-producing animals. Recently, a number of studies have demonstrated that no ingested foreign DNA was found in the blood or tissues of poultry [17, 20] and livestock [35] fed with transgenic grains. Similar results were also obtained from studies with other animal species, such as fish [36] and rabbits [37]. A study suggested that the transgenic DNA of GM soya was not detectable as it moved through the GIT of healthy humans [38]. Ma *et al*. also reported that *phyA2* genes were not found in the blood, heart, liver, spleen, kidneys and breast muscle of laying hens fed with a PTC diet [15]. The results from the present study were consistent with the above findings, which showed that none of the transgenic gene (*phyA2*) was detected in any broiler tissues of the GM group.

### Detection of phyA2 protein in digesta and tissues of broilers

In the present study, the *phyA2* protein was detected in 100% of PTC and PTC diet samples, 70% of duodenum samples and 30% of jejunum samples of broilers fed with PTC, but not detected in the lower gastrointestinal tract (ileum and rectum) samples. Similar evidences in laying hens were provided by Ma *et al*. [15]: the *phyA2* protein was found in the crop (100%), gizzard (87.5%), duodenum (37.5%) and jejunum (12.5%), and not found in the ileum, cecum, and rectum of laying hens fed with PTC. These results also are consistent with the results obtained from laying hens [39]. However, Chowdhury *et al*. [16] demonstrated that Cry1Ab protein was detected at very high concentrations in rectal digesta of pigs. The possible explanation for degradation of the recombinant protein might be that the ingested protein is usually degraded into small peptides and/or free amino acids, which are ingested in the upper intestine [40].

The differences concerning the digestive fate between the DNA and protein in the intestine have been obtained also by Ma *et al*. [15] and Walsh *et al*. [41] This might be because extraneous protein has a steadier structure than its gene and could be protected against rapid degradation in the upper GIT.

Proteins produced from foreign genes might be undetectable in the tissues of animals due to their rapid degradation in the gut. Ash *et al*. [39] demonstrated that CP4 EPSPS protein was not detected in the liver, egg, or feces of laying hens fed with Roundup Ready (RR) soybean meal. Similar results were obtained also in some studies on GM corn. The Cry1Ab protein was not detected in the breast muscle of broilers fed with transgenic corn (MON 810) either [42]. Furthermore, the *phyA2* protein was not found in the blood, heart, liver, spleen, kidney, and breast muscle from laying hens fed with the PTC diet [15]. To date, the appearance of transgenic protein in tissues of broilers fed with GM crops has not been reported in studies with broilers. The current results showing that no *phyA2* protein was detected in the heart, liver, kidney, breast and leg muscles of broilers are consistent with those previous studies.

In addition, in the present study, we did not measure phytate P, non-phytate P, and phytase activity in the digesta of broilers. However, we got some relative information from the studies of Nyannor *et al*. [43] and Gao *et al*. [13]. In their studies, it was reported that the addition of corn expressing phytase to the control diet increased residual phytase activity and decreased phytic acid P concentration in the digesta of proventriculus and gizzard, jejunum, and ileum of broilers and laying hens. It was also observed that residual phytase activity and phytic acid P concentration in the digesta decreased caudally along the GIT. Therefore, we speculate that the similar cases might have occurred in our present study.

## Conclusions

In summary, this study shows that PTC had no adverse effect on intestinal microflora of broilers. The *phyA2* DNA was not detected in the intestinal digesta of broilers. The transgenic *phyA2* protein was detected in the digesta of the duodenum and jejunum, but not in the ileal and rectal digesta. *PhyA2* DNA or protein was not transferred to the heart, liver, kidney, breast and leg muscles of broilers. These results collectively indicate that the incorporation of PTC in the broiler diets should not be an issue in terms of the accumulation of foreign phytase DNA and proteins in different organs, as well as their influences on intestinal microflora.

## Supporting Information

S1 File The ARRIVE Guidelines Checklist. (PDF)
